# Good Natured People

**Published:** 1875-06

**Authors:** 


Good-Natured People.—The specially social quality is good nature, amiability, the desire to please, the kindness of heart that avoids giving offence, and can not bear to hurt any one's feelings. A good-natured person may frankly disagree with you, but he never offends. He quarrels good-naturedly. He boxes with gloves on. When he fences ever so deftly, there is a great soft button on the end of his foil. He may satirize, ridicule, open up all your weaknesses and absurdities, but so kindly that you cannot kelp loving him. He can not say a harsh, hard, bitter, or contemptuous thing, because he has no hardness and no contempt. This is simple, natural goodness, like the goodness of fond and friendly animals. It may not be a high moral virtue; there is no particular merit in it any more than in beauty or any natural gift; but it is a very delightful quality, and those who do not possess it should imitate those who do. Just as we avoid in person, dress, or manners, anything that may give disgust or pain, so must we do in our conversation. We must no more use vulgar expressions than we would wear vulgar garments. Our talk should be as clean as our fingers. We should no more bite one with our words than with our teeth. An angry word is as bad as a blow, and a satirical word is like a sting. If we are never to say anything *to* a person which will give him disgust or pain, we must be even more careful not to say anything *of* any one which will injure him in the estimation of others. Playful, good-natured criticism upon the little foibles and peculiarities of others may be harmless, and even useful, but it ceases to be good-natured when it gives pain. Slander is a sin much worse than theft. Charity forbids that we should even tell the truth, when that truth can wound and injure. The best rule is to say all the good we can of every one, and to refrain from ever saying evil, unless it becomes a clear matter of duty to warn some one against him.

